# Factors associated with future intentions to use personal vaporisers among those with some experience of vaping

**DOI:** 10.1111/dar.12574

**Published:** 2017-05-31

**Authors:** Bernice Hua Ma, Hua‐Hie Yong, Ron Borland, Ann McNeill, Sara C. Hitchman

**Affiliations:** ^1^ School of Population Health University of Melbourne Melbourne Australia; ^2^ Department of Public Health La Trobe University Melbourne Australia; ^3^ Nigel Gray Fellowship group Cancer Council Victoria Melbourne Australia; ^4^ Department of Addictions, Institute of Psychiatry, Psychology, and Neuroscience King's College London London UK; ^5^ UK Centre for Tobacco and Alcohol Studies Nottingham UK

**Keywords:** personal vaporiser, e‐cigarette, attitude, past experience, intention

## Abstract

**Introduction and Aims.** Personal vaporisers (PV), including e‐cigarettes, may be a harm reduction strategy for tobacco control. This study aims to identify factors associated with future intentions to vape among smokers and ex‐smokers in Australia and the UK. **Design and Methods.** Cross‐sectional data of smokers and ex‐smokers (n = 1199, mean age = 45.3 years, 44.8% male), collected in 2014/2015 and divided into four subgroups: smoking past vapers (SPV), smoking vapers (SV), ex‐smoking past vapers (ESPV) and ex‐smoking vapers (ESV), from the International Tobacco Control Australia and UK surveys were analysed by using regression models. **Results.** Higher vaping satisfaction increased vaping intentions for all groups except ESPV. Perceiving PVs as less harmful predicted intentions to vape for all groups except ESV. The importance of PVs for stopping smoking predicted lower intentions to continue vaping for SV, but higher intentions to initiate vaping for SPV. The importance of PVs for cutting down smoking was a positive predictor only for SPV. Among ex‐smokers, importance for maintaining not smoking was a positive predictor for ESPV, but not for ESV. The importance of perceiving vapour being less harmful also depended on vaping status for ex‐smokers. The only country interaction was that only in the UK was perceiving PVs as less harmful associated with intention among SPV. **Discussion and Conclusion.** Factors influencing intentions vary by smoking and/or vaping status, with greater differences between the ex‐smoker subgroups. This is consistent with PVs being seen as a way of managing smoking, rather than something that has intrinsic value, for all except the ex‐smoking vapers. [Ma BH, Yong H‐H, Borland R, McNeill A, Hitchman SC. Factors associated with future intentions to use personal vaporisers among those with some experience of vaping. Drug Alcohol Rev 2017;00:000–000]

## Introduction

Personal vaporisers (PV), also known as e‐cigarettes, are relatively new products that have generated huge controversy. Some argue that they may play a central role in speeding the elimination of smoking 1, 2; others are concerned that they may appeal to many non‐smokers who would not otherwise use nicotine or, in the case of ex‐smokers, return to smoking 3. This paper is concerned with factors that might motivate smokers and ex‐smokers who are either: (i) past vapers (with some experience of use) to take vaping up; or (ii) current vapers to continue vaping. The paper is restricted to two countries, Australia and the UK, which have markedly different regulatory environments around these products.

Awareness and use of PVs is growing internationally, with the levels of use in each country apparently influenced by the prevailing societal response towards them 4. In Australia, there is no specific nation‐wide law addressing the regulation of PVs. Instead, laws relating to therapeutic goods, poisons and tobacco control apply to PVs under different circumstances. Vaping with nicotine is prohibited except with a doctor's prescription. By contrast, UK has welcomed a harm reduction framework. Under the European Union Tobacco Products Directive 5 implemented in May 2016, England has moved towards regulating PVs largely as consumer products with limits on levels of nicotine. Limited advertising is allowed, largely local, such as at point of sale, and sales to under 18‐year‐olds are prohibited 6. Based on the International Tobacco Control (ITC) survey, current use of PVs in Australia increased from 0.6% in 2010 to 6.6% in 2013. By contrast, in the UK, current use rose from 4.5% to 18.8% respectively 4. More recent data from the UK showed prevalence of use increased further from 16.8% in early 2013 to 21.6% in early 2016 7.

People evaluate whether to perform a behaviour based on both the instrumental and experiential components of the given behaviour 8, 9. Research has identified five major instrumental reasons for vaping: first, to help quit smoking or avoid relapse 10, 11, 12, 13, 14, 15, 16, 17, 18, 19, 20 and second, to deal with cravings and withdrawal symptoms 10, 11. One study found that 90% of vapers reported that it provided relief from cravings 10. Third, they are perceived to be less harmful than ordinary cigarettes 10, 11, 12, 13, 15, 16, 21, 22. One study found that 70.3% of the respondents who were aware of PVs perceived them to be less harmful than traditional cigarettes 21; however, this belief has declined in some countries, such as UK 23. Fourth, some vape to reduce their cigarette consumption 11, 15, 18, 19, 24. Fifth, a small proportion choose PVs because they are cheaper (realistic especially for the refillable tank types) [10,14]. The other major reason to vape is experiential—the satisfaction they experience from use (desired psychoactive effects), complemented by more pleasant taste and smell 18.

There is little research relating reasons for use to intentions to continue use or to future use. Social 25 and temporal comparison 26 theories propose that individuals tend to evaluate their attitudes by using reference points, such as current behaviour or expectancies from their social group. More generally, Context, Executive, and Operational Systems theory 9 proposes that behavioural choices are made through a combination of beliefs about the value of the behaviour for them and direct effects, especially affective reactions to the behaviour, the latter being modulated by beliefs about those reactions. Where the two sets of influences are congruent, current behaviour patterns are likely to be sustained, and if they shift in tandem, there will be a rapid change in behaviour. This analysis is based on the principle that we tend not to do things that we do not perceive or experience any value for, and thus, behaviours that persist must have value for the person, if only the immediate affective experiences of use. However, when there are conflicting motivations, as there are for smoking, attempts to change behaviour are likely to fail.

Based on the analysis in the preceding texts, we predict that interest in future use is likely to vary by smoking status, with ex‐smokers who do not currently vape being unlikely to be interested in use, except to prevent relapse. Current non‐vaping smokers are predicted to compare vaping to smoking when deciding if they will vape in the future. Similarly for current users, smokers are likely to compare vaping to smoking in deciding whether to continue vaping. Vaping is also predicted to have its potential as a quitting aid, or at least a means of limiting smoking consumption, being strongly associated with continued use. Vaping ex‐smokers are predicted to want to continue if they value the experiences of use or to sustain abstinence from smoking. Finally, we also examined if the different policy environments in Australia and the UK would impact on both types of intention by testing for any by country interactions.

## Methods

### ITC study design

Data come from one wave of the Australian and UK arms of the ITC Four Country (ITC‐4) survey. The ITC‐4 study is conducted via a mix of telephone and web‐based methods. Data were collected between September 2014 and February 2015. The longitudinal study recruits smokers aged 18 years and older, but retains those who subsequently quit. The initial sample and replenished samples up to 2012 were recruited by using the random digital dialling method, and some of the new participants from the latter waves (2013 and 2014 surveys) were recruited by phone from a single source probability‐based panel via an address‐based frame. Detailed descriptions of the ITC‐4 conceptual framework 27 and methods 28 are available elsewhere.

### Participants

From 2941 surveyed, 1199 participants who had ever tried using PVs were included in this study. They were divided into four subgroups based on smoking and vaping status: 480 smoking vapers (SV), 549 smoking past vapers (SPV), 88 ex‐smoking vapers (ESV) and 82 ex‐smoking past vapers (ESPV). Current vapers were defined as those who currently use an e‐cigarette at all, even as low as less than weekly.

### Measures

The questions we asked used ‘e‐cigarette’ as the generic descriptor (as we believed it was the most used term at the time), but it was defined first and specified that it included e‐shisha and PVs.

### Outcomes

#### Intentions to use PVs in future/continue to use

For current vapers, future intention to use was assessed by using the question ‘Do you plan to keep on using e‐cigarettes, or do you plan to stop using sometime in the foreseeable future?’, with a binary response of ‘(1) keep using’ versus ‘(0) plan to stop sometime in the foreseeable future’; for past vapers, intention was assessed by using the question ‘Assuming that you have the opportunity, how likely are you to use e‐cigarettes in the future?’, with a 5‐point scale response option ranging from ‘(5) definitely yes’ to ‘(1) definitely not’. The wording of the questions was designed to eliminate lack of availability as a possible reason.

### Predictors

#### Past experiences and belief about vaping

Four questions (see Table S1 for actual wording) were used to assess past experiences of vaping: level of satisfaction derived from PVs relative to ordinary cigarettes [rated on a 5‐point scale from (1) totally unsatisfying to (5) more satisfying], negative side effects related to PV use [(2) yes/(1) no], number of friends and family using PVs daily [from (1) none to (4) four or more] and use of PVs inside home [(2) yes/(1) no]. A single question assessed perceived harmfulness of PVs relative to regular cigarettes, rated on a 5‐point scale from a lot less to a lot more harmful, dichotomised into ‘(1) less harmful’ versus ‘(0) no less harmful’ (including equal and don't know) for analysis.

#### Importance of reasons for using PVs in future/continue to use

Twelve questions assessed the importance of both experiential (Questions 6, 7 and 16 in Table S1) and instrumental (Questions 8–15 and 17) reasons for deciding to use PVs in the future, either continuing to use or taking vaping up, rated on a 5‐point scale from (1) not at all important to (5) extremely important. Questions about using PVs in smoke‐free areas, for stopping smoking and for cutting down smoking were asked only of smokers (Questions 10–12), while those about using PVs as a way of not smoking and to stop from returning to smoking were asked only of ex‐smokers (Questions 13–14).

Based on correlations, two pairs of variables, ‘safety’ and ‘less harmful’ (*r* = 0.67, see Table S1), and ‘not returning to smoking’ and ‘not smoking’, were combined by averaging their scores to yield ‘less harmful’ (Table 2) and ‘help to stay quit or prevent relapse’ (Table 4). The importance of stopping smoking and cutting down smoking were also very highly correlated (*r* = 0.86), but were not combined on theoretical grounds: Cutting down is more likely to imply persisting dual use if it predicts differentially to stopping.

### Control variables

Age, sex, country, cohort, survey mode (phone vs. Internet), ever daily use of PVs, heaviness of smoking index (for smokers) and quit attempts in the last 6 months (for smokers).

### Data analyses

All analyses used stata 14. Group differences were examined by using analysis of variance for continuous variables and χ^2^ tests for categorical ones. In the main analysis, the four subgroups were analysed separately. Logistic regression models were employed to predict intention to keep using in SV and ESV, a binary outcome, while linear regression models were used to predict intention to use (a continuous outcome measure) in SPV and ESPV. Bivariate regression analyses were initially performed on each of the set of key questions, followed by multivariate analyses that adjusted for potential confounders (control variables) (M1) to examine motives independently associated with the intention to use, and intention to keep using, PVs in the future. These were then followed by backward stepwise regression analyses (M2) to identify the most significant independent associates of intention to use and intention to keep using PVs, but only including those with a *P*‐value for effect of less than 0.3 in order to reduce the number of variables in the stepwise regressions. In addition, due to the relatively small sample of ex‐smokers, the control variables that were included were those with a *P* < 0.3 level of association with the outcome.

## Results

Detailed characteristics are presented in Table 1. The four subgroups differed by education, income, country and survey mode. Among vapers, smokers were more likely to intend to continue PV use than ex‐smokers. Similarly, among past vapers (only 34% with daily experience; >1 week), smokers were even more interested in PV use than ex‐smokers. It is notable that in the two past‐vaper groups, only a small minority expressed any intention to vape again, and among the vapers, only a minority were interested in continuing, less among the ex‐smokers.

**Table 1:** Characteristics of respondents who have ever used personal vaporisers by smoking and/or vaping subgroups

Variables	SV (*n* = 480)	SPV (*n* = 549)	ESV (*n* = 88)	ESPV (*n* = 82)
*Country (%)* *				
Australia	31.3	45.5	15.9	47.6
UK	68.8	54.5	84.1	52.4
*Age in years*				
Mean (SD)	45.0 (12.9)	45.2 (13.5)	45.6 (11.3)	44.6 (13.2)
*Gender (%)*				
Male	45.0	44.4	50.0	40.2
Female	55.0	55.6	50.0	59.8
*Education (%)* *				
Low	37.3	36.8	40.9	36.6
Medium	29.2	27.7	28.4	30.5
High	26.7	21.3	26.1	23.2
No information	6.5	14.2	4.6	9.8
*Income (%)* *				
Low	48.5	44.6	43.2	24.4
Medium	22.7	24.6	27.3	30.5
High	21.9	21.7	21.6	29.3
No information	6.9	9.1	7.8	15.9
*Survey mode (%)* *				
Web	76.7	62.1	70.5	84.2
Phone	23.3	37.9	29.5	15.8
*Intention to continue to vape (%)* *				
Yes	39.2	NA	27.3	NA
No	60.8	NA	72.7	NA
*Intention to take up vaping (%)* *				
Definitely yes	NA	7.7	NA	1.2
Probably yes	NA	16.9	NA	1.2
Might or might not	NA	27.7	NA	13.4
Probably not	NA	21.7	NA	28.1
Definitely not	NA	26.0	NA	56.1

* Significant subgroup differences at *P* < 0.05; ESPV, ex‐smoking past vapers; ESV, ex‐smoking vapers; NA, not applicable; SPV, smoking past vapers; SV, smoking vapers.

There were large by‐group differences in possible motives (Table 2). As might be expected, past vapers had generally lower scores on both the experience and importance measures than vapers. Among vapers, the ex‐smoker group generally had more positive attitudes and placed greater importance in the various reasons than the current smokers. Among past vapers, there were few differences between the two smoker groups. The only clear exception was that the importance of satisfaction with vaping was higher in smoking past vapers than ex‐smoking past vapers. This contrasts with the finding for vapers where ex‐smokers were more likely to endorse it than the smokers.

**Table 2:** Means (SD) and percentages of predictor variables by smoking and/or vaping subgroups

Variables	SV (*n* = 480)	SPV (*n* = 549)	ESV (*n* = 88)	ESPV (*n* = 82)	Group difference (overall *P*‐value)
*Past experiences and belief about PVs*					
Level of satisfaction	2.51 (0.89)	2.13 (0.83)	3.20 (1.05)	2.22 (0.86)	a, b, c, d, f (<0.001)
Side effect (% yes)	11.9	10.2	9.1	8.5	(0.689)
Friends and family using	2.19 (1.03)	1.83 (0.97)	2.63 (1.15)	1.51 (0.92)	a, b, c, d, f (<0.001)
Use at home (%)	67.5	NA	84.1	NA	b (0.002)
Perceived less harmful (%)	72.1	60.7	85.2	58.5	a, b, c (<0.001)
*Importance of reason for deciding to use*					
Satisfying	3.28 (1.10)	3.27 (1.34)	3.53 (1.12)	2.51 (1.33)	b, c, e, f (<0.001)
Less smelly	3.24 (1.28)	2.75 (1.37)	3.57 (1.44)	2.90 (1.38)	a, b, c, d, f (<0.001)
Friends using	1.79 (1.12)	1.40 (0.87)	1.58 (1.03)	1.51 (0.92)	a, c (<0.001)
Manage stress	3.13 (1.26)	2.91 (1.39)	2.95 (1.28)	2.22 (1.19)	a, c, e, f (<0.001)
Less harmful	3.59 (1.10)	3.41 (1.26)	4.16 (0.98)	3.12 (1.43)	a, b, c, d, f (<0.001)
Relative harm of second‐hand vaping	3.54 (1.20)	3.23 (1.31)	3.76 (1.14)	3.09 (1.27)	a, c, d, f (<0.001)
Use in smoke‐free area	2.89 (1.38)	2.45 (1.36)	NA	NA	a (<0.001)
Help to stop smoking	3.51 (1.24)	3.28 (1.40)	NA	NA	a (0.005)
Help to cut down smoking	3.56 (1.19)	3.21 (1.34)	NA	NA	a (<0.001)
Help to stay quit	NA	NA	4.26 (1.07)	2.72 (1.46)	f (<0.001)

ESPV, ex‐smokers not currently vaping; ESV, ex‐smokers currently vaping; NA, not applicable; SPV, smokers not currently vaping; SV, smokers currently vaping. Statistically significant group comparison: a = 1 versus 2; b = 1 versus 3; c = 1 versus 4; d = 2 versus 3; e = 2 versus 4; f = 3 versus 4.

### Predicting intention to keep using PVs among SVs

Predictors of intention to continue vaping among SV are presented in Table 3. Higher level of satisfaction with vaping was positively related to intention to continue using PVs in all analyses, with the effect remaining strong in the combined model, as was perceiving PVs to be less harmful than conventional cigarettes and the importance of being able to use PVs in smoke‐free areas. By contrast, the importance of using PVs to stop smoking was negatively associated with continuing to use in the future.

**Table 3:** Predictors of intention to use personal vaporisers (PV) for smokers currently vaping (SV) and smokers not currently vaping (SPV)

Predictors	Intention to keep using among SV (*n* = 480)			Intention to use among SPV (*n* = 549)		
	Regression models, OR (95% CI)			Regression models, Β (SE)		
	Bivariate	M1	M2	Bivariate	M1	M2
*Past experiences and belief about PVs*						
Higher level of satisfaction	1.46 (1.18, 1.80)**	1.60 (1.19, 2.15)**	1.55 (1.17, 2.05)**	0.54 (0.06)**	0.41 (0.06)**	0.41 (0.06)**
Side effect yes	0.97 (0.55, 1.72)	1.11 (0.55, 2.23)	—	−0.31 (0.18)	−0.42 (0.15)**	−0.42 (0.15)**
Friend and family using	1.07 (0.90, 1.28)	0.98 (0.79, 1.22)	—	0.20 (0.05)**	0.17 (0.05)**	0.17 (0.05)**
Use at home	1.94 (1.29, 2.93)*	1.43 (0.87, 2.36)	1.45 (0.89, 2.38)	NA	NA	NA
Perceived less harmful	2.36 (1.51, 3.67)**	2.03 (1.20, 3.46)**	2.16 (1.29, 3.60)**	0.66 (0.11)**	0.30 (0.10)**	0.29 (0.10)**
*Importance of reason for deciding to use*						
Satisfying	1.18 (1.00, 1.40)	1.05 (0.83, 1.32)	—	0.29 (0.07)**	0.15 (0.04)**	0.15 (0.04)**
Less smelly	1.16 (0.99, 1.34)	1.08 (0.87, 1.32)	—	0.17 (0.04)**	−0.02 (0.04)	—
Friends using	0.97 (0.82, 1.14)	0.92 (0.74, 1.14)	—	0.14 (0.06)*	0.07 (0.05)	00.07 (0.05)
Manage stress	1.14 (0.98, 1.32)	1.02 (0.83, 1.26)	—	0.21 (0.04)**	0.01 (0.04)	—
Less harmful	1.11 (0.92, 1.34)	1.10 (0.82, 1.48)	—	0.32 (0.05)**	−0.07 (0.06)	—
Relative harm of second‐hand vaping	1.04 (0.89, 1.21)	0.92 (0.73, 1.17)	—	0.23 (0.04)**	−0.01 (0.04)	—
Use in smoke‐free areas	1.32 (1.15, 1.51)**	1.38 (1.17, 1.64)**	1.37 (1.17, 1.61)**	0.15 (0.04)**	−0.02 (0.04)	—
Help to stop smoking	1.03 (0.89, 1.20)	0.75 (0.55, 1.01)	0.75 (0.56, 0.99)*	0.40 (0.03)**	0.23 (0.06)**	00.23 (0.06)**
Help to cut down smoking	1.21 (1.03, 1.41)*	1.28 (0.93, 1.75)	1.34 (0.98, 1.79)	0.40 (0.04)**	0.11 (0.06)*	00.11 (0.05)*

Note: M1 is regression model adjusted for age, sex, country, cohort, survey mode, ever used PV daily, heaviness of smoking index and recent quit attempts; M2 is backward stepwise regression model adjusted for the same set of variables as M1.

* *P* < 0.05;

** *P* < 0.01; —, predictor not selected in the stepwise regression model. CI, confidence interval; NA, not applicable; OR, odds ratio

B, beta coefficient; SE, standard error.

### Predicting intention to use PVs among SPVs

There were more independent predictors of intention to vape for SPV than SV (Table 3). Similar to SV, both higher vaping satisfaction and perceiving PVs to be less harmful were positively related to intention to use in the future. Having experienced any side effects of PV use in the past was associated with no future intention to use PVs. Having friends or family members using PVs was associated with increased interest in future use, as was the importance of PVs being satisfying. Unlike for SV, for SPV, the importance of using PVs to stop smoking and to cut down smoking was positively related to intention to use in the future.

### Predicting intention to keep using PVs among ESVs

Table 4 shows that none of the past experience and belief measures predicted intention to continue using PVs for ESV. However, the importance of satisfaction with vaping was positively related, while the importance of how much less harmful second‐hand vapour is was associated with lesser intentions to continue.

**Table 4:** Predictors of intention to use personal vaporisers (PV) for ex‐smokers currently vaping (ESV) and ex‐smokers not currently vaping (ESPV)

Predictors	Intention to keep using among ESV (*n* = 88)			Intention to use among ESPV (*n* = 82)		
	Regression models, OR (95% CI)			Regression models, Β (SE)		
	Bivariate	M1	M2	Bivariate	M1	M2
*Past experiences and belief about PVs*						
Higher level of satisfaction	1.38 (0.87, 2.19)	1.21 (0.53, 2.76)	—	0.11 (0.11)	0.003 (0.12)	—
Side effect yes	1.69 (0.37, 7.67)	0.54 (0.06, 4.92)	—	−0.38 (0.34)	0.03 (0.38)	—
Friend and family using	1.25 (0.82, 1.91)	1.27 (0.65, 2.48)	—	0.20 (0.10)*	0.11 (0.13)	0.11 (0.09)
Use at home	2.54 (0.52, 12.30)	0.77 (0.06, 34.11)	—	NA	NA	NA
Perceived less harmful	2.28 (0.47, 11.16)	1.29 (0.06, 28.16)	—	0.28 (0.19)	0.39 (0.23)	0.36 (0.17)*
*Importance of reason for deciding to use*						
Satisfying	1.97 (1.21, 3.21)**	3.95 (1.44, 10.81)**	3.13 (1.51, 6.51)**	0.10 (0.07)	−0.04 (0.09)	—
Less smelly	1.04 (0.75, 1.45)	1.42 (0.74, 2.73)	—	0.08 (0.07)	0.09 (0.12)	—
Friends using	0.84 (0.51, 1.38)	0.64 (0.28, 1.48)	0.67 (0.38, 1.19)	0.19 (0.10)	0.16 (0.13)	0.19 (0.09)*
Manage stress	1.12 (0.77, 1.62)	0.89 (0.47, 1.70)	—	0.11 (0.08)	0.02 (0.11)	—
Less harmful	1.05 (0.59, 1.88)	0.50 (0.18, 1.40)	—	0.15 (0.07)*	0.13 (0.11)	—
Relative harm of second‐hand vaping	0.80 (0.53, 1.19)	0.38 (0.17, 0.87)*	0.47 (0.27, 0.82)**	−0.03 (0.08)	−0.21 (0.10)*	−0.19 (0.08)*
Help to stay quit or prevent relapse	1.60 (0.87, 2.89)	1.06 (0.37, 3.06)	—	0.17 (0.07)*	0.14 (0.10)	0.19 (0.08)*

Note: M1 is regression model adjusted for age, sex (past vapers only), country, cohort, survey mode (past vapers only), ever used PV daily (vapers only); M2 is backward stepwise regression model adjusted for control variables which have a *P*‐value smaller than 0.3 in Model 1.

* *P* < 0.05;

** *P* < 0.01; —, predictor not selected in the stepwise regression model. CI, confidence interval; NA, not applicable; OR, odds ratio

B, beta coefficient; SE, standard error.

### Predicting intention to use PVs among ESPVs

Table 4 shows that perceiving vaping to be less harmful was positively associated with intention to vape in future for ESPV as were the importance of friends using and the importance of PV use to help remain a non‐smoker. By contrast, the importance of how much less harmful second‐hand vaping is was negatively associated.

### Country interactions

The only significant by‐country interaction was for perceiving PVs to be less harmful than smoking in the SPV group (*P* < 0.001). We repeated our analyses for the SPV group stratified by country (Table S2). This showed that the overall significant effect of relatively lower perceived harm of PVs as an influence on future intentions occurred only in the UK with no association for Australia.

## Discussion

The results of this study showed that there is limited interest in taking up vaping again among past users, and most current vapers plan to stop using. The factors potentially influencing future use of PVs varied by both smoking and vaping status, but not always in the ways we expected. None of the measures were consistently associated with intentions in all four groups, but importance of help to stop smoking was strongly related for both smoker groups.

For smokers currently vaping, higher experienced satisfaction with vaping and perceiving PVs to be less harmful than conventional cigarettes were positive predictors of intentions. This is consistent with previous findings that vapers were more likely to believe PVs to be less harmful than conventional cigarettes as compared with past vapers 29. Further, the more important they think stopping smoking as a reason for using PVs is, the less likely they plan to continue using PVs in the future. This suggests that those who value the use of PVs to quit smoking generally see their use as temporary, while the minority who intend to continue to vape may be less concerned about smoking cessation. The finding of a positive relationship between perceived satisfaction with vaping and intention to continue use suggests that as PVs become engineered to better deliver nicotine 30, interest in continued use may rise.

For smokers not currently vaping, past experiences predicted future intentions. As expected, greater satisfaction with vaping, perceiving them to be less harmful than smoking, the importance of vaping satisfaction and of their role in quitting and cutting down were all associated with intentions to vape again. Taken together, the findings also suggest that use is more likely if they are shown to be superior cessation aids and are made more generally satisfying 10. The only by‐country interaction for this group was of the belief that PVs are less harmful, being positively associated with intentions in the UK, but not in Australia. There is no ready explanation for this country difference. However, if it is real, it may be that the issues of access and greater uncertainty about harm in Australia might have masked the importance of risk perception as a determinant of intention to use PV.

For ex‐smokers currently vaping, experienced satisfaction with vaping relative to smoking was not related to intentions, while the importance of satisfaction with vaping itself was. This suggests that once people have given up smoking and taken up vaping, the comparative experienced satisfaction of the two behaviours is no longer important and what determines future intentions to continue the current behaviour is its intrinsic value. Interestingly, the importance of PV vapour being less harmful to people around them was negatively related to intention. It may be that this reflects making comparisons between vaping and not using any nicotine product at all (rather than with smoking), so if they perceived vaping as having any level of harm, then it would discourage continued use. It seems unlikely to us that believing second‐hand vaping to be relatively harmless would inhibit use.

Turning now to ex‐smokers not currently vaping, believing PVs to be less harmful than ordinary cigarettes predicted future intention as did several of the importance measures. The latter includes friends using and help to stay quit, both positively associated, while relative harms of second‐hand vapour to others were negatively related as they were for ex‐smoking vapers. This latter finding strengthens our argument that this question is being interpreted as concern that there might be harm from exposure to second‐hand vapour, not literally as a response to ‘How much less harmful the vapour is to others around you’. Taken together, it suggests that concerns about harm to others from second‐hand exposure may be inhibiting some who might otherwise use, but more may be willing to take it up if it is seen as helpful for staying quit.

This study has several limitations. First, we use country as a proxy for the different policy environments, and by‐country differences could be caused by many other factors. However, as we only found one interaction, suggesting that neither is particularly important. This means that we can more confidently generalise our findings to comparable countries and a wide range of policy environments. Second, our study relies on cross‐sectional comparisons that are usual in studies of intention. Intention is logically the consequence of past experiences, but it can affect how a person recalls and describes their past experiences, so we cannot rule out the possibility that the intentions, once formed, reshaped reported beliefs and reasons for intending. Further, we did not study those with no experience of vaping who may be less likely to intend to use than those with past experience. Another limitation is the relatively small sample size for ex‐smokers and the smaller samples of vapers from Australia, both reducing the power to find any, but the large effects in these sub‐groups. Finally, there were some differences in group composition between those completing the survey by phone as compared with online. We are not sure what these differences mean. Older and lower SES respondents were more likely to be surveyed by phone, but this does not explain the differences found. We cannot think of any way that the differential group membership by survey mode we found could have led to any of the by‐group differences we found, but cannot rule them out.

It is important to note that we only studied intentions, and they do not always translate into behaviour. Longitudinal studies, or in this case additional waves of this study, are needed to see to what extent intentions are predictive of use. That said, all had had some experience of use, so the responses can be assumed to be grounded in at least some past experience, albeit it is less clear for past users, as some had no regular use and might have used less attractive early generation devices.

In summary, the findings of this study indicate that among those with at least some experience of vaping, both current and former users, intentions around vaping appear to be largely rational. The misperceptions that exist around harms 23 may be inhibiting use. Misbeliefs may also be leading to less quitting smoking that would otherwise be the case. Evidence shows that vaping is at least as effective as conventional nicotine replacement therapy for smoking cessation 31, 32 and that it is much more acceptable to smokers 33 and that newer products may be even more effective in nicotine delivery 34, 35 and satisfying. Further, given the rated importance of satisfaction, it is likely that there will be increased use of these products if levels of satisfaction generated by these products increase. Whether increased satisfaction and thus more extended use lead to reduced relapse or just persistent recreational use is unclear at this point. However, our finding that ex‐smoking past vapers saw preventing relapse as a reason they might use in the future and that only a minority intended to continue for recreational reasons both suggest that the role of vaping in smoking cessation is more important for most.

## Conclusions

Factors influencing intentions to use PVs vary by smoking and/or vaping status. The findings are generally consistent with evidence from previous studies suggesting that people evaluate whether to perform a behaviour based on both the instrumental and experiential components of the given behaviour 8 and translate these beliefs into intentions in a largely rational way. It remains unclear the extent to which intentions will translate into behaviour, which we acknowledge is also strongly affected by affective reactions 9. The likelihood of intentions translating into action is also likely to be affected by changes in the PV products and the nature of the social context, including rules and emergent norms around PV use. However, based on the present levels, it is unlikely that there will be a strong move towards vaping among past users and most current vapers plan to stop. If services decided to promote vaping to facilitate smoking cessation, work would be needed to change the minds of non‐vapers to encourage them to try it as a means of helping them quit. At present, there seems little risk of a mass movement towards vaping.

## Supporting information


**Table S1.** Predictors of intention to use or intention to keep on using personal vaporisers
**Table S2.** Predictors of intention to use personal vaporisers (PV) among smokers not currently vaping by country
