# Supplementary material for: Factors associated with future intentions to use personal vaporisers among those with some experience of vaping
Source: Drug Alcohol Rev. 2017 May 31;37(2):216–25. doi: 10.1111/dar.12574 (PMC5798997; doi:10.1111/dar.12574)
Supplement: Supplementary file 1 — Table S1. Predictors of intention to use or intention to keep on using personal vaporisers Table S2. Predictors of intention to use personal vaporisers (PV) among smokers not currently vaping by country [file DAR-37-216-s001.docx]

**Appendices**

**Table S1.** **Predictors of intention to use or intention to keep on using personal vaporizers**

| Questions | Short name | Scale | Subgroup |
| --- | --- | --- | --- |
| **Past experiences and belief about PV use** | | | |
| 1. As compared to ordinary cigarettes, how would you rate the satisfaction you get from e-cigarettes? | Level of satisfaction | 1-5  (1. Totally unsatisfying – 5. More satisfying than ordinary cigarettes) | All |
| 2. Do you think electronic cigarettes are more harmful than regular cigarettes, less harmful or are they equally harmful to health? | Perceived less harmful | 0-1  (0:no less harmful, 1: less harmful) | All |
| 3. Have you experienced negative side effects that concerned you, even if temporary, that you think might be related to your use of e-cigarettes? | Side effect | 1-2  (1. No; 2. Yes) | All |
| 4. Among your friends and family, how many, if any, do you know who use e-cigarettes regularly? | Friends and family use | 1-4  (1. None; 2: only 1 person; 3. 2 or 3; 4. 4 or more) | All |
| 5. Do you use e-cigarettes inside your home? | Use at home | 1-2  (1. No; 2. Yes) | Current  vapers |
| **Importance of reasons for deciding to use PVs in future/continue to use** | | | |
| To what extent, if at all, are the following important to you, as reasons for using e-cigarettes? (For people who are using e-cigarettes)  To what extent, if at all, are the following likely to be important to you, in deciding whether to use e-cigarettes in the future?(For people who are not currently using e-cigarettes) | | | |
| 6. How satisfying they are? | Satisfying | 1-5  (1.Not at all important –2.slightly important-3.somewhat important-4.very important- 5.extremely important) | All |
| 7. They don’t smell as bad as cigarettes? | Less smelly | As above | All |
| 8. How safe they are to use? | Safety | As above | All |
| 9. How much less harmful they are than smoking? | Less harmful | As above | All |
| 10. That you can use them in places where you can’t smoke ordinary cigarettes? | Use in smoke-free area | As above | Smokers |
| 11. As a way to stop smoking ordinary cigarettes? | Stop smoking | As above | Smokers |
| 12. As a way of cutting down on ordinary cigarettes? | Cut down smoking | As above | Smokers |
| 13. As a way to stop you from returning to smoking ordinary cigarettes? | Not return to smoking | As above | Ex-smokers smokers |
| 14. As a way of not smoking ordinary cigarettes? | Not smoking | As above | Ex-smokers smokers |
| 15. How they help you manage stress? | Manage stress | As above | All |
| 16. How many of your friends use them? | Friends using | As above | All |
| 17. How much less harmful the vapour is to others around you? | Relative harm of second-hand vaping | As above | All |

**Table S2. Predictors of intention to use personal vaporizers (PV) among smokers not currently vaping by country**

|  | **Bivariate** | | **M1 Multivariate Regression** | | **M2 Stepwise Regression** | |
| --- | --- | --- | --- | --- | --- | --- |
| **Predictors** | **Intention to use**  **β(SE)** | | **Intention to use**  **β(SE)** | | **Intention to use**  **β(SE)** | |
|  | **UK**  **(n=299)** | **Australia**  **(n=250)** | **UK**  **(n=294)** | **Australia**  **(n=244)** | **UK**  **(n=294)** | **Australia**  **(n=244)** |
| **Past experiences and beliefs about PV use** | | | | | | |
| Higher level of satisfaction | 0.59** (0.09) | 0.50** (0.08) | 0.40** (0.08) | 0.40** (0.08) | 0.43** (0.08) | 0.41** (0.08) |
| Side effect - Yes | -0.36 (0.23) | -0.31 (0.28) | -0.59** (0.18) | -0.21 (0.24) | -0.58** (0.18) | -0.20 (0.25) |
| Friend and family use | 0.13 (0.07) | 0.28** (0.10) | 0.13* (0.06) | 0.20* (0.09) | 0.13* (0.06) | 0.21* (0.09) |
| Perceived less harmful | 0.82** (0.15) | 0.44** (0.15) | 0.57** (0.14) | 0.04 (0.14) | 0.54** (0.13) | 0.07 (0.14) |
| **Importance of reason for deciding to use PV in the future** | | | | | | |
| Satisfying | 0.30** (0.05) | 0.25** (0.05) | 0.14* (0.06) | 0.16* (0.06) | 0.15** (0.06) | 0.15* (0.06) |
| Less smelly | 0.11* (0.05) | 0.23** (0.05) | -0.12* (0.05) | 0.19** (0.07) | – | – |
| Friend using | 0.12 (0.08) | 0.16 (0.10) | 0.05 (0.07) | 0.10 (0.09) | 0.06 (0.07) | 0.09 (0.09) |
| Manage stress | 0.23** (0.05) | 0.17** (0.05) | 0.02 (0.05) | -0.02 (0.06) | – | -- |
| Less harmful | 0.37** (0.06) | 0.26** (0.06) | -0.07 (0.08) | -0.11 (0.09) | -0.08 (0.08) | -0.11 (0.08) |
| Vapour less harmful to people around | 0.26** (0.05) | 0.18** (0.06) | 0.05 (0.06) | -0.05 (0.07) | – | – |
| Use in smoke-free areas | 0.22** (0.05) | 0.08 (0.05) | 0.08 (0.05) | -0.17** (0.06) | – | – |
| Help to stop smoking | 0.43** (0.05) | 0.36** (0.05) | 0.22** (0.07) | 0.30** (0.09) | 0.22** (0.07) | 0.26** (0.09) |
| Help to cut down smoking | 0.41** (0.05) | 0.38** (0.05) | 0.10 (0.07) | 0.05* (0.07) | 0.12 (0.07) | 0.07 (0.09) |

Note: M1 regression on each variable adjusted for age, sex. country, cohort, survey mode, ever used PV daily, heaviness of smoking index and recent quit attempts; M2 is backward stepwise regression model; adjusted for the same set of variables as M1.**P* <0.05; ***P* <0.01; – predictor not selected in the stepwise regression model.
